# Representation, activism, health promotion, and communication: The role of art in advancing global health and social justice

**DOI:** 10.1371/journal.pgph.0004761

**Published:** 2025-07-23

**Authors:** Mark Donald C. Reñosa, Kelly E. Perry, Siddharth Srivastava, Angeli Rawat, Zaida Orth, Phuong Bich Tran, Diane Woei-Quan Chong, Joseph Kazibwe, Maira Shaukat, Germán Andrés Alarcón Garavito, Mazen Boroudi, Vivek Dsouza, Shahreen Chowdhury, Bachera Aktar, Daniela Da Costa, Daniela Ochaita, Kerry Scott

**Affiliations:** 1 Heidelberg Institute of Global Health, University of Heidelberg, Heidelberg, Germany; 2 Department of Epidemiology and Biostatistics, Research Institute for Tropical Medicine, Muntinlupa, Philippines; 3 Bloomberg School of Public Health, Johns Hopkins University, Baltimore, Maryland, United States of America; 4 Nicholas School of the Environment, Duke University, Durham, North Carolina, United States of America; 5 Department of Health Policy and Management, Texas A&M University, College Station, Texas, United States of America; 6 School of Population and Public Health, University of British Columbia, Vancouver, Canada; 7 The James Black Gallery, Vancouver, Canada; 8 School of Public Health, University of the Western Cape, Cape Town, South Africa; 9 Nuffield Department of Primary Care Health Sciences, University of Oxford, Oxford, United Kingdom; 10 Center for Health Services Research, Institute for Health Systems Research, National Institutes of Health, Ministry of Health Malaysia, Shah Alam, Malaysia; 11 Department of Clinical Sciences, Lund University, Malmö, Sweden; 12 Faculty of Medicine, Universidad de los Andes, Bogota, Colombia; 13 Department of Epidemiology and Global Health, Umeå University, Umeå, Sweden; 14 Department of Social Welfare, Bukkyo University, Kyoto, Japan; 15 Edward J. Bloustein School of Planning and Public Policy, Rutgers University, New Brunswick, New Jersey, United States of America; 16 Institute of Public Health, Bengaluru, India; 17 Liverpool School of Tropical Medicine, Liverpool, United Kingdom; 18 BRAC James P Grant School of Public Health, BRAC University, Dhaka, Bangladesh; 19 Unit of Medical Anthropology, Center of Health Studies, Universidad del Valle de Guatemala, Guatemala City, Guatemala; 20 INCAP Research Center for the Prevention of Chronic Diseases, Institute of Nutrition of Central America and Panama, Guatemala City, Guatemala; 21 School of Global Health, Faculty of Health, York University, Toronto, Ontario, Canada; University of Washington Bothell, UNITED STATES OF AMERICA

## Abstract

This viewpoint advocates for the inclusion of art in global health discourse and practice. We explore four areas in which art can be leveraged to improve global health: (1) to amplify disenfranchised voices, (2) to advance social justice activism, (3) to strengthen communities and individuals, and (4) to improve global health communication. Drawing on community-driven art initiatives, we argue for an inclusive approach that respects diverse cultural perspectives and uplifts marginalized voices. Emphasizing interdisciplinary collaboration and ethical engagement, our framework invites global health discourse and practice to integrate art in order to foster empathy, challenge systemic inequities, and envision sustainable futures. By centering art, we seek to enrich the global health discipline with insights and transformative potential grounded in human experiences, cultural diversity, and shared humanity.

## Introduction

Understanding the importance of our shared humanity lies at the heart of global health [1–3]. The universal values of shared humanity [4]—empathy, compassion, solidarity, equity, justice, collaboration, and cultural respect—benefit global health by fostering effective changes and sustainable improvements in health [3]. Yet, amidst the quest for meaningful change, one crucial element has been underappreciated in global health: the integration of art. Art, in its myriad forms—*visual* (painting, sculpture, photography, street art, digital media, crafts, caricatures), *performance* (dance, theater, circus, music, stand-up comedy), *literary* (poetry, prose, storytelling), or *other artistic expressions* (art installations, virtual reality experiences)—can illuminate our shared humanity and evoke emotional connections beyond borders and social divisions.

**Fig 1:**
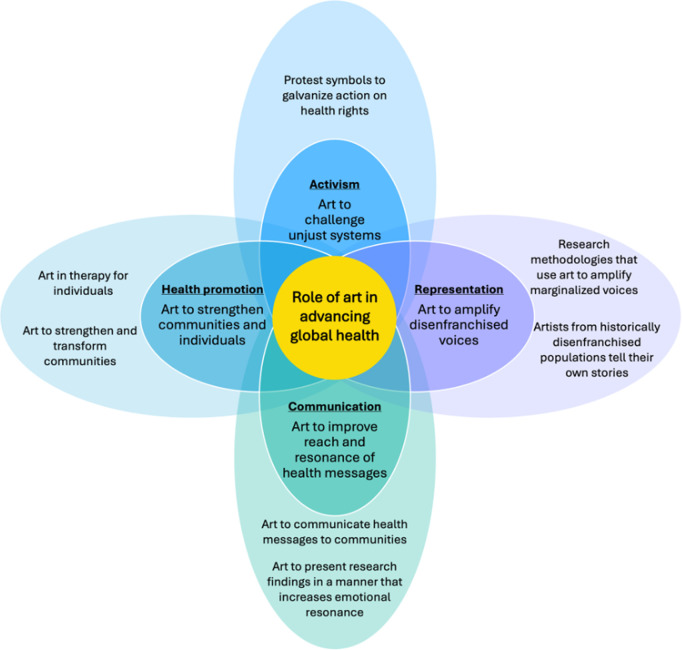
Roles of art in advancing global health.

In this viewpoint, we—alumni of the Emerging Voices for Global Health (EV4GH) program [5]—argue that increasing the use of art in global health discourse, research, and practice can: (1) amplify perspectives and insights from disenfranchised populations, (2) advance social justice activism, (3) promote health by strengthening communities and individuals, and (4) improve global health communication (**Fig 1**). Across these areas, we suggest that art can play a valuable role in decolonizing global health by improving the representation of diverse needs and aspirations of our global community, and particularly by elevating the voices, experiences, and knowledge of historically marginalized populations. We conclude with reflections and recommendations on how global health actors can use art within inclusive global health discourse, research and practice.

### 1. Representation: Art to amplify disenfranchised voices

Art can elevate voices that have historically been relegated to the sidelines of global health conversations. Artists from marginalized populations offer unique insight into personal, social, and political forces in their communities. Outsiders who pay close attention to art produced by these artists will gain more nuanced and contextualized understanding of health realities. In addition to the work of professional artists, all people can use art to express themselves and tell their own stories. Global health actors can offer art-based research methods, such as photovoice [6] or body mapping [7], to enable marginalized people to produce art that illuminates their voices and experiences.

These mechanisms align with the decolonizing global health agenda by challenging hegemonic power structures and narratives [8]. Amplifying the art made by historically marginalized populations and using art in global health research encourages richer, more inclusive dialogue, deepens emotional understanding and connection across populations, and shifts the power to tell one’s story and share one’s expertise from dominant, high-income country actors to historically marginalized people. Having sovereignty over the narrative about one’s life, experiences, and ideas increases the possibility to also have sovereignty over the proposed solutions.

For example, photovoice studies in Nigeria [9], Uganda, [10] and Mozambique [11] have empowered community members to highlight health challenges and co-design solutions. In Nigeria, individuals with neglected tropical diseases described the physical and psychosocial impacts of conditions like leprosy and lymphatic filariasis, leading to the co-design of healing-focused support groups [9]. In Uganda and Mozambique, the combination of photos and narratives revealed daily challenges and amplified marginalized voices, demonstrating how visual methods can elucidate underlying social determinants of health [10,11]. Similarly, street photography projects, such as Humans of New York by Brandon Stanton [12] and Being Human by Lee Fennings [13] serve to humanize individuals from diverse backgrounds, fostering empathy and highlighting the intrinsic value and dignity of every person, regardless of their social or cultural differences. Theater has also played a significant role in this context, with plays like The Vagina Monologues opening discourses about women’s reproductive health, sexuality, and sexual abuse, eventually evolving into the ‘V-Day movement’, which seeks to end violence against women and girls [14]. Drag is a performance art that playfully and powerfully explores gender expression through costumes, make-up, and exaggerated personas. It has become a symbol of liberation and resilience, serving as a platform to raise awareness about health and social issues that disproportionately affect LGBTQIA+ communities [15,16].

### 2. Activism: Art to advance social justice activism

Art can advance activism through its “transgressive capacity to reveal what has been otherwise hidden or silent” [17]. This capacity has enabled art to serve as a powerful tool for unifying social movements and challenging injustice. Art has been used in activism to speak truth to power and to unite people with disparate perspectives to recognize their common humanity, thereby increasing the resonance of social justice demands.

**Fig 2:**
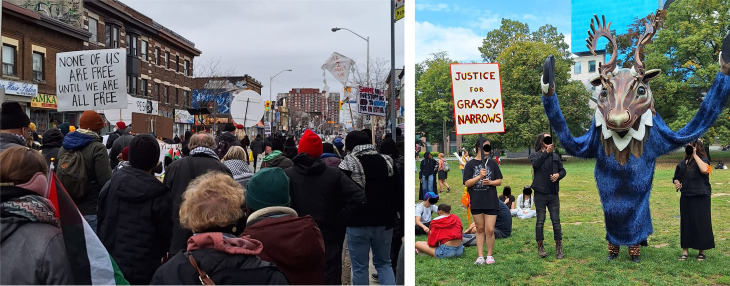
Art in activism. LEFT: A home-made kite with a line from Alareer’s poem “If I Must Die” can be seen held above a pro-Palestine protest in Toronto on January 28, 2024. RIGHT: An activist dressed as a deer at the Grassy Narrows River Run in Toronto on September 18, 2024, a protest for the Grassy Narrows Indigenous community’s ongoing fight for justice after their water was poisoned by mercury dumping. Images courtesy of Kerry Scott (co-author).

Art has been crucial in activism on a wide range of health issues including the AIDS crisis, the opioid epidemic in the US, air pollution, and the genocide in Gaza. Art has played a strong role in HIV/AIDS activism in the 1980s and 1990s, including the Treatment Action Campaign’s use of performance such as die-ins [18] and the NAMES Project AIDS Memorial Quilt [19]. More recently, Nan Goldin’s performances, photography, and documentary films have exposed the perpetrators of the opioid crisis in the US, drawing a stark connection between their profits with the pain, suffering, and loss faced by those affected [20]. Thanawat Maneenawa and the Thailand Clean Air Network‘s art installation exhibit at the Bangkok Arts and Cultural Center called attention to the injustices of air pollution and united individuals behind the right to clean air [21,22]. Poet Refaat Alareer’s poem “If I Must Die” has played a potent role in pro-Palestine activism from 2023 to date [23]. This poem expresses a wish to live beyond death as a source of hope for children in Gaza. It conjures the image of a homemade kite – popular across Palestine – carrying forward this message. Alareer was killed on December 6, 2023, but his words exemplify *sumud* (steadfastness) [24] – a Palestinian epistemology of resistance that predates colonial conceptualizations of ‘activism’ – and lives on, written on homemade kites that served as protest signs across demonstrations in the US, Canada, and Europe demanding an end to Israel’s destruction of Gaza, including the bombing of hospitals and killing of healthcare workers (**Fig 2**). Large scale puppetry has also been used in protest movements [25,26], including for Indigenous land rights in Canada (**Fig 2**).

Global health researchers and practitioners must recognize the long history of art in indigenous and resistance movements, while critically examining colonial histories of extractive ‘activism’ [27,28] and harness its power to expose and challenge global systemic issues, drive justice and reconciliation conversations, highlighting our shared humanity.

### 3. Health promotion: Art to strengthen communities and individuals

Art can support health promotion [29] through facilitating individual healing and community level empowerment. Art promotes healing and empowerment by enabling people and communities to tell their own stories, grapple with hidden truths, process trauma, introspect, and reimagine in ways that can serve as a pathway toward healing. When designed to engage participants or communities as active agents of change, art is a powerful tool for perceiving and analyzing social, political, and economic structures and acting against oppressive systems [30,31]. Creative methods enable individuals and communities to explore, understand, and challenge social injustices, allowing them to redress power dynamics and express their perspectives on challenging topics, such as mental health, violence, and stigma.

Art therapy has shown strong potential to help people process trauma of war [32–34], social isolation and depression [35–39], and discrimination [40], and has enabled men and boys to reimagine masculinity in ways that reject violence and misogyny [41,42]. Feminist art has enabled individuals of all genders to examine and realize their personal and communal potential, subverting traditional norms and enhancing community resilience [43,44]. Comedy, as an artistic tool, has proven valuable in health promotion interventions [45,46] and can enable health professionals to address challenging topics like racism, homophobia, and white privilege within their work [47,48]. Participatory theater, such as Boal’s Theatre of the Oppressed and Augusto Drama, music, and street theater have been used to raise awareness about health issues and prompt communities to approach their challenges in new ways [49]. In Ethiopia, the Fendika Cultural Center uses arts (cross-cultural music, dance, and visual arts engagement) to promote mental health and cultural unity, while celebrating cultural heritage [50]. This is especially important given the context of rising ethnic tensions and conflict in Ethiopia.

**Fig 3:**
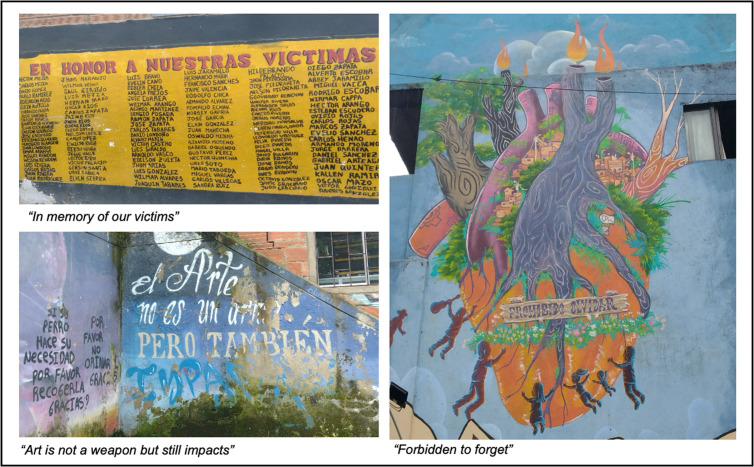
Street Arts in Medellin, Colombia. Images courtesy of Germán Andrés Alarcón Garavito and Daniela Ochaita (co-authors).

Art has also been part of a broader process of social, economic, and political rejuvenation and transformation. For instance, in Medellín, Colombia, communities took their collective experience of violence and resilience, and rewrote it through arts (i.e., wall murals, graffiti, dance and rap performances), echoing the potency of arts in strengthening their communities (**Fig 3**) [51–58].

Interest in using art for improving health and well-being is increasing. In 2019, the World Health Organization (WHO) highlighted the role of arts in promoting health equity, preventing illness, and treating chronic conditions across the lifespan [59]. Building on this, the Jameel Arts & Health Lab, in collaboration with the The Lancet and WHO, released a series of articles in the Lancet Global Health that strengthens the evidence base for the role of arts in addressing non-communicable diseases [60]. While these efforts are promising, decentering them from Global North hegemonies through integration of Ubuntu [61] and Buen Vivir [62] health philosophies in decolonial frameworks is essential to ensure they drive meaningful impact. For example, *Ubuntu!* Fest organized by the Decolonial Arts Centre, centers the African philosophy of *Ubuntu* through performance, storytelling, and visual art by Black women artists, fostering collective healing and enhancing mental wellbeing through relationality, shared humanity, and decolonial aesthetics [63]. In global health education, the Art and Global Health Center at University of California, Los Angeles employs art-based interventions grounded in human rights and social justice principles [64]. Similarly, Universidad Andina Simón Bolívar (Ecuador) has made efforts to integrate *Buen Vivir* and *Ubuntu* into health planning and curricula, emphasizing ecological balance, interdependence, and co-production of knowledge [65]. In Latin America more broadly, community-based art practices inspired by *Buen Vivir*—including participatory murals and street theater—have addressed health-related themes such as environmental justice, food sovereignty, and intergenerational trauma.

### 4. Communication: Art to improve the reach and resonance of global health messages

Art can enhance the communication of public health messages and research findings. Global health actors can utilize art as a tool for public health engagement, knowledge mobilization and dissemination, influencing change across socio-ecological levels – from individual health behaviors to broader cultural narratives and policies. [66]

For health communication, art enables bidirectional dialogue that respects communities as holders of embodied knowledge, not passive message recipient. In Bangladesh, Entertainment-Education dramas have been used in national health campaigns for behavioral change communications on reproductive and maternal health [67], child immunization, mental health [68] and oral rehydration therapy building upon South Asian oral and folk traditions such as *jatra* [69,70]. Similarly, creative campaigns such as ‘know your lemon [71]’, which uses fruits to raise awareness breast cancer signs, offer refreshing and accessible approaches to health messaging. Art was also instrumental during the Ebola epidemic in Liberia, wherein the song ‘Ebola in Town’ inspired storytelling, radio dramas, and comic books to communicate targeted and culturally appropriate health messages. In Malawi [72] and South Africa [73], music, dance and photography disseminated health messages on malaria, HIV, substance abuse, and gender-based violence to stimulate dialogue and mobilize change.

**Fig 4:**
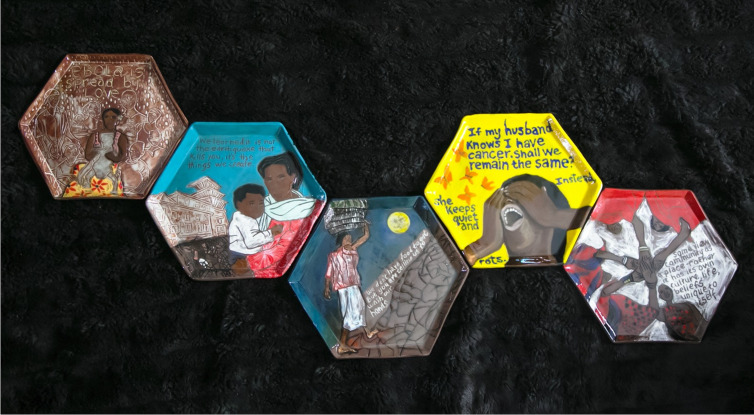
When words turn into stone. Image courtesy of Angeli Rawat (co-author).

Art can amplify research findings related to the experiences of historically marginalized populations and increase the emotional resonance of these findings. For example, Hurley and colleagues created a theatre performance called the “COVID Monologues” to translate qualitative research findings on the impact of the COVID-19 pandemic on diverse segments of American society [26]. Global health researcher and artist Angeli Rawat developed a ceramic art installation based on research in Liberia, Nepal, Ethiopia, and Uganda that explored the personal impact of Ebola and cancer; the discordance between aid donors and receivers; the structural drivers of health; and community engagement. This work distilled words and concepts from research into emotionally compelling art, while integrating contextual significance and reflexivity (**Fig 4**) [74].

## Actions to increase the use of Arts in Global Health

Despite great potential, integrating art in global health remains insufficient and few researchers, educators, and practitioners receive any formal training in the use of art within global health. **Table 1** presents guidance for global health actors (researchers, educators, and practitioners) who wish to increase the use of art in their global health work.

**Table 1:** Increasing the use of art in global health: Recommendations for researchers, educators, and practitioners.

Actors	Recommendations
All people engaged in global health work	• **Engage with and promote art** produced by people from marginalized communities ◦ Read books, watch films, explore visual art, etc. made by artists from historically oppressed populations ◦ Amplify the work of artists from marginalized communities across platforms
Researchers	• **Use art in global health research and dissemination**, where appropriate ◦ Community-based participatory methods including photovoice and body mapping can be compelling art-based research methodologies ◦ Develop interdisciplinary research centers and networks that bring together experts from the arts, humanities, social sciences, and health fields to collaborate on art and health equity research and practice ◦ Partner with performance artists in dissemination activities • **Work with ethics boards to support the ethical use of art in research** ◦ Partner with ethics boards to ensure art-based methods prioritize the dignity, rights, and lived experiences of participants • **Study the impact of using art** in health promotion and communication ◦ Explore and evaluate how art and art-based interventions influence health, such as through emotional expression, social connection, or physical activity ◦ Develop new research methods and tools to assess the impact of art-based interventions on health and well-being • **Promote the use of art in health policies and programs** ◦ Conduct policy analysis and advocacy research to identify opportunities and barriers to integrating art and culture into health policies and programs ◦ Synthesize evidence on the health benefits of art engagement to inform policy and practice recommendations ◦ Develop and test innovative funding models and partnerships that can sustain art and health initiatives over the long term ◦ Compensate Indigenous knowledge holders using FAIR CARE principles [75] for artistic intellectual property
Educators	• **Train students on how to use art in global health** ◦ Teach students how to use of art in health promotion, health communication, global health activism and to amplify marginalized voices ◦ Incorporate community-engaged learning opportunities for students to work with artists on health-related projects ◦ Create opportunities for students from diverse disciplines (e.g., public health, art, psychology, nursing) to collaborate on art and health research and practice projects ◦ Foster critical thinking and reflexivity among students about the power dynamics and ethical considerations involved in using different art forms for health promotion ◦ Develop continuing education programs for health professionals on how to use arts-based methods in their practice ◦ Provide mentorship and support for students and early career researchers who are interested in pursuing careers at the intersection of art, culture, and health equity • **Use art to teach about global health** ◦ Spotlight art produced by marginalized people to better understand diverse life experiences ◦ Publicize art made by marginalized individuals to convince powerholders to change in some way (e.g., screening documentaries, sharing TikTok videos, showing paintings made by historically marginalized people at global health events)
Practitioners	• **Use art-based methods to engage communities** in health education, behavior change, and social mobilization activities ◦ Collaborate with artists and other partner organizations on arts-based health activities and messages that are engaging, culturally relevant, and accessible to diverse participants ◦ Provide ongoing training and support for health professionals to develop skills in using arts-based methods for health promotion and patient care ◦ Forge partnerships with arts institutions and funders to scale up and sustain arts-based global health initiatives • **Integrate art to improve clinical healthcare** ◦ Integrate art and cultural activities into clinical care and treatment programs to enhance patient experiences and outcomes • **Use art to strengthen health policy** ◦ Use art-based methods to amplify the voices and experiences of marginalized communities and advocate for policy and systems change

## Barriers and tensions in integrating art into global health action

Achieving meaningful integration of art into global health requires addressing barriers and avoiding potential pitfalls. Resource limitations are a major barrier given that art interventions are often discounted and seen as expendable [29,76]. Resources can only be secured for the long-term if we reframe art as a valuable component of global health efforts, supported by evidence of impact through robust evaluation [29,76]. Researchers, practitioners, and educators may also encounter a lack of institutional support and poor networking infrastructure between art and health [29,76]. These gaps can be reduced by integrating art into medical and public health curricula, formalizing relationships between artists and practitioners, and developing mechanisms for cross-sectoral exchange.

After securing funding and institutional support, researchers, practitioners, and educators must be careful to avoid missteps and errors during the implementation phase. For instance, those attempting to use art in other global health work taking place outside their cultural context must work closely with local community members to develop relevant and enriching art opportunities, and to avoid imposing external, potentially harmful, art forms. Even with the best intentions, the use of art in global health can be tokenistic or shallow if it does not truly empower communities. Art may even end up replicating ‘parachute research’ rather than sustaining transgenerational indigenous art traditions. Short-term projects or externally curated outputs may only serve to reinforce the very hierarchy’s art seeks to deconstruct.

Additional ethical issues within art-based work in global health include ensuring participant consent to engage with art or have their art shown to others, the risk of art being used to misrepresent communities or individuals, the potential of re-traumatization through art, and protecting the rights of local artists. There are powerful actors who could benefit from co-opting or commercializing artistic movements—to colonize grassroots representations for the sake of profit, branding, or advocacy. This kind of appropriation and violence can not only dilute the intent and cultural significance of art but also redirect benefits outside the communities that created them and cause harm (e.g., re-traumatization) to the communities. Sustained and close engagement with ethicists, elders, and community members is essential to ensure that arts-based work respects the dignity and well-being of participants.

Artistic movements in global health face acute risks of corporate and institutional co-optation, where grassroots symbolism is commodified for specific agendas. This mirrors patterns observed in environmental justice grantmaking, where foundations funding often reshapes movement priorities while maintaining extractive power dynamics [77]. The platform cooperativism movement demonstrates how even artistic initiatives risk absorption into market logics if they uncritically adopt entrepreneurial frameworks [78]. Similarly, modern graffiti’s transition from protest art to real estate marketing tool exemplifies how aesthetics loses their emancipatory potential when divorced from community ownership [79]. To resist this, it behooves the global health community to focus on community leadership, local sovereignty, and ownership of artistic endeavors so that these arts are imagined, directed, and led by local communities. Principles for ethical engagement—such as obtaining permission to reproduce, protecting narrative agency and control in line with FAIR CARE principles are key to strike a balance between artistic intellectual property remaining with originating communities and ethical dissemination [80].

At a broader level, artistic movements also face critical tensions that reveal inherent contradictions between its emancipatory aims and the structural realities of institutional engagement. One persistent challenge lies in the respectability paradox, where marginalized communities must negotiate between preserving radical authenticity and securing policy gains. Historical analyses of Norwegian fetish activists’ 1990s HIV prevention campaigns illustrate this tension vividly. While their sanitized BDSM imagery successfully challenged psychiatric stigmatization and built bridges with public health authorities, it simultaneously obscured the subculture’s transgressive roots [81,82]. These dynamics echo broader patterns in LGBTQIA+ health advocacy, where demands for dignity often necessitate conforming to heteronormative frameworks, raising ethical questions about whose expressions of identity are deemed “safe enough” for mainstream acceptance. Another tension emerges through temporal myopia, where memorialization risks neutralizing art’s urgent political charge. The AIDS Memorial Quilt’s trajectory exemplifies this paradox [19,83]. Initially conceived as a radical act of communal grief and rage during the height of the epidemic, its institutionalization as a federally archived relic has gradually muted its confrontational power [83,84]. Recent scholarship critiques how the Quilt’s physical fragmentation and digital preservation have transformed it from a living protest tool into a depoliticized historical artifact, distancing contemporary audiences from the ongoing realities of HIV/AIDS disparities [85]. This phenomenon mirrors broader concerns about how art-based memorial projects may inadvertently signal resolution rather than sustaining pressure for systemic change [83]. These unresolved tensions do not negate art’s transformative potential but rather highlight the need for reflexive praxis. They demand frameworks that center enduring partnership models, resist the neutralization of radical aesthetics, and prioritize community sovereignty over short-term policy wins.

Ultimately, the transformative power of the art in global health will depend on a willingness to push for truth, equity, and power-sharing. Through the deliberate consideration of barriers and anticipated risks, as well as the central inclusion of community members at each step, art-informed approaches can fulfill their potential to build empathy, break down systemic inequities, and contribute to global health and social justice in a range of settings around the world.

## Author reflexivity

As EV4GH fellows, we leverage our diverse backgrounds and experiences to invite nuance to discourse on and praxis within the decolonization of global health. The impetus for exploring art arose during our EV4GH venture in Colombia in 2022, where we observed the influence of historical colonial practices on local healthcare systems and the resulting artistic forms of expression that bred healing and unity within communities.

We acknowledge that we have received higher educations in both low- and middle-income countries and/or high-income countries, with some of us currently pursuing PhDs and/or research-related careers in high-income countries. Some of us have received the privilege of scholarships to pursue higher education. Additionally, some of us are involved in health policy and systems research within governments and non-government organizations in the Global South and/or our respective home countries.

Given our positionalities and affiliations with academic institutions in high-income countries, we acknowledge that we may be complicit in and have benefited from the inherent (neo)colonial power structures within global health and use such positionalities to critique persistent inequities in current systems toward a world where health equity and justice exists for all peoples.

## Conclusion

Increasing the use of art in global health can strengthen global health activism, improve health promotion, amplify historically oppressed voices, and bolster the communication of research findings and health messages. Art has the power to integrate evidence with empathy, increase public understanding of health issues, and galvanize communities and political leaders toward action. Global health researchers, educators, and practitioners, can benefit from art’s capacity to offer profound insights into human dignity and transcend geographical, temporal, and hierarchical boundaries. Art can be integrated with science to humanize global health research, practice, and teaching and to dismantle long-held inequities in global health.
